# Overview of the brand journey and opportunities for future studies

**DOI:** 10.1007/s43039-023-00069-0

**Published:** 2023-03-13

**Authors:** Sandra Maria Correia Loureiro

**Affiliations:** Business Research Unit (BRU-IUL), ISCTE – Instituto Universitário de Lisbon, Av. Forças Armadas, 1649-026 Lisbon, Portugal

**Keywords:** Brand, Identification, Connection, Symbolism, Integration, Behavioral

## Abstract

With this study, brand managers can have an overview of the major concepts and characteristics of brands over time, while academics receive a mapping of the most analyzed topics and suggestions for future research. Based on the documents published in Scopus and Web of Science databases using the word “brand”, this article aims to provide an overview of the brands and suggest opportunities for future research. Text mining clustering allowed the processing of a large amount of information and organized the first overview of the concepts that have been studied. Thus, first, we examine the existing definitions of a brand. Then, we provide a historical perspective of the topics associated with brand constructs and their associations and present a framework for the psychological characteristics of the branding process. Finally, we present the future trends.

## Introduction

*Originally the term *“*Brand*” referred to a piece of burning wood. In the Middle Ages, it becomes a verb meaning a permanent mark with a hot iron. In the seventeenth century, it represented a mark of ownership (Ries & Ries, 2005). Yet, it is in the late nineteenth and twentieth centuries that it grows in relevance and started to designate goods, services, and institutions, but now also destinations, people, and robots. Brands are present in human lives in the physical, online, or virtual world (Loureiro et al., 2021).

Brands and studies on brands have rapidly evolved over time and there is a need to map such evolutions to guide both researchers and practitioners. Other reviews discuss some particular aspects of branding by focusing on specific concepts—for instance, authenticity (Nunes et al., 2021), engagement (Bilro & Loureiro, 2020), love (Gumparthi & Patra, 2020), or human brand (Osorio et al., 2020)—or are devoted to a certain context (e.g., Bhattacharya & Sen, 2003; Hollebeek et al., 2014), period of time (Górska-Warsewicz & Kulykovets, 2020; Barros-Arrieta & García-Cali, 2021; García-Cali), or are dedicated to the global versus local symbolism of the brand (Liu et al., 2021), lacking a more holistic perspective of brands. Because brands and their concepts are a core topic in marketing, it is relevant to aggregate the knowledge to offer a summary view on the topic.

In this vein, the goal of this article is to bring together the research on branding to understand its roots and evolution and identify gaps. We intend to provide an overview of the brand journey—presenting theory, themes, context, psychological characteristics, and methodology over the different decades—and suggesting opportunities for future research. An overview of the brand concepts across different decades provides academics and managers with a temporal perspective of how brands have been analyzed, perceived, and managed. For such purpose, this article seeks to answer the following research questions: What are prominent concepts of the brand in the different decades? What major research gaps are found and how could future research evolve from now on?

To do so, the paper is structured as follows. First, we present the data extraction process and give the first description of the studies and the main concepts from the text mining clustering. Second, we identify keywords in the different decades of research on branding and discuss concepts and brand models proposed and analyzed in prior studies. Finally, we set out the future trends organized as gaps, theory, themes and context, psychological characteristics, and methodology. This article helps academics to shape future research and gives key brand models and tools to practitioners, contributing to a better knowledge of how to manage their brands.

## Data extraction and analysis

Two academic databases—aggregating several publishers, as Elsevier, Wiley, Taylor and Francis, Emerald, or Sage—were used to detect relevant documents to expose an overview of the evolution of brand concepts: Scopus and Web of Science (WoS). The search for documents was performed on 2 January of 2022, using the word “brand”. Time limitations were not considered.

**Fig. 1:**
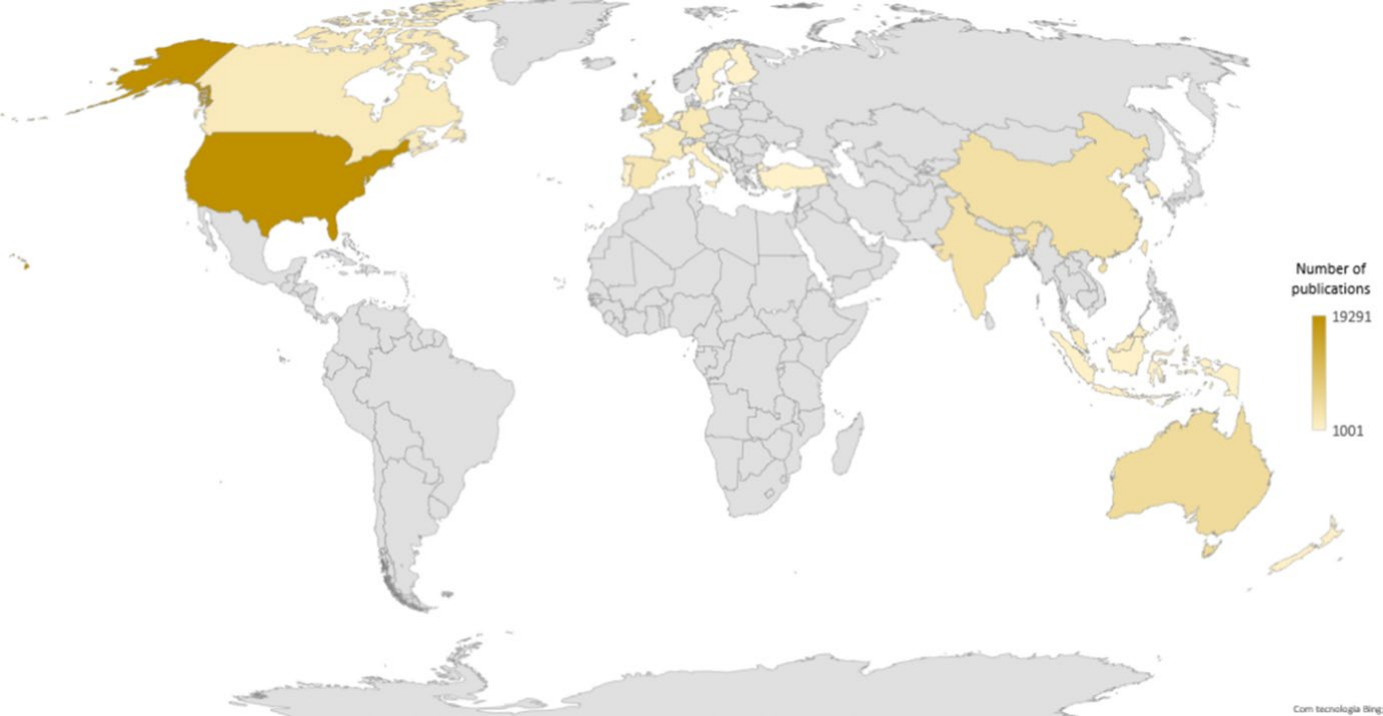
Distribution of articles by territory, according to the corresponding author. *Note*: Considered only countries with more than 1000 documents. Twenty-two countries were considered with 1000 or more documents (articles)

The initial number of documents from Scopus was 104, 595 (75,176 articles) and 108,484 (78,571 were articles) from WoS. When merging and eliminated duplication 80, 738 remain. Overall, the article publications are mostly from the United States of America (U.S.A.) (19,291; 24%), followed by the United Kingdom (U.K.) (7959; 10%) and Australia (5287; 7%), China (4451; 6%) and India (4132; 5%) (see Fig. 1). In the European Union, Spain (3045; 4%) and Germany (2906; 4%) are the dominant countries. Based on the affiliation of the correspondent author it was possible to understand the origin of the published articles. Thus, Anglo-Saxon countries tend to be the most prolific, followed by Asians, which may demonstrate that their respective affiliated universities may be the ones that most drive publications in this area and may also offer better conditions for them to be conducted.

The last decades of twentieth century are largely dominated by U.S.A. and U.K. In the twenty-first century Asian countries, such as China, India, and Malaysia, start to gain relevance. The same occurs in Europe with Greece, Spain, even Turkey, or New Zealand in Oceania.

**Fig. 2:**
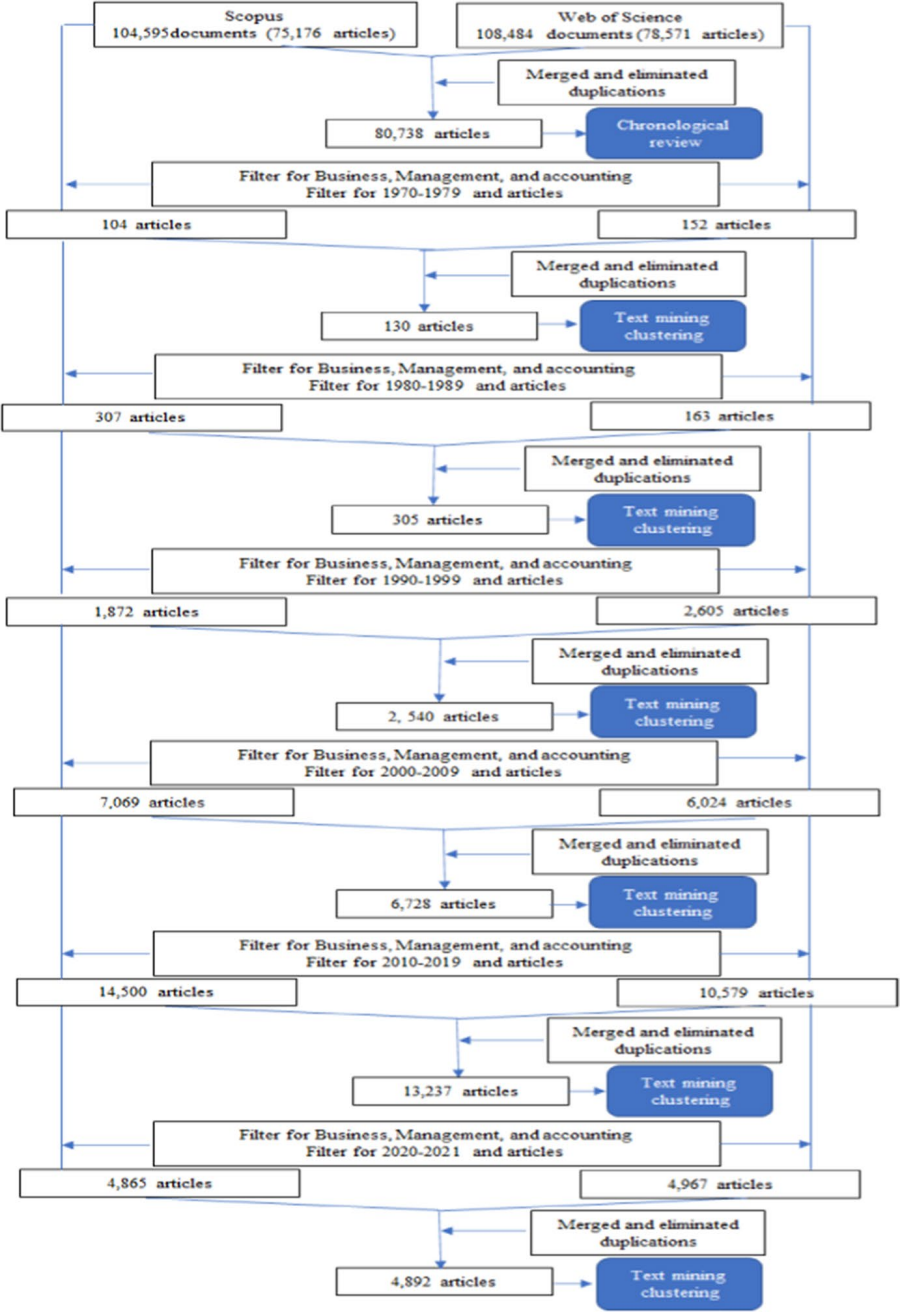
Methodological process

When filtering for Business, Management, and accounting, considering only articles and regarding the different decades in both databases, it is possible to obtain –through text mining clustering—the clusters for each decade in analysis (see Fig. 2).

Text mining clustering was performed to get a first overview of the concepts that have been studied. The text clustering aggregates unstructured text to extract relevant clusters. The MeaningCloud text mining tool (Meaningcloud, 2022) can analyze the text of the papers and create clusters, each one representing text that is similar (Spinakis & Chatzimakri, 2005; groups (clusters) by analyzing the text of each paper (Fan et al., 2006). In this first overview, only papers published in journals were analyzed, only concepts were considered, and the stop words inserted were 2000 (see Table 1).

**Table 1:** Text clustering

Cluster	Score	Associated cluster	Score
1970’s			
Brand loyalty	200.59	Purchasing behaviour	149.34
		Purchase decision	26.40
Brand advertising	180.89	Television advertising	180.89
		Promotion	36.94
		Choice behaviour	90.63
		Brand name	256.29
Customer satisfaction	138.67	Attitude	36.00
1980’s			
Brand choice	189.30		
Market segmentation	77.24	Product category	172.29
		Market share	73.18
CRM		Customer's perceptions	72.63
		Claims	43.39
Quality assurance/control		Evaluation	56.21
		Quality‐assuring mechanisms	188.66
		Quality and price	54.85
		Product quality	36.58
		Brand quality	15.01
1990’s			
Customer/brand loyalty	127.54	Loyal customers	131.02
Perceived quality	142.42		
Brand equity	85.63		
Customer attitude/attitude toward the brand	73.70		
Relationship quality	252.60		
Perceived value	116.20		
E-communication	201.91		
Perceived risk	24.79		
2000’s			
Corporate social responsibility	64.16		
Brand image	135.28		
Brand experience	40.28		
Service recovery	29.40		
Perceived value	104.67		
Brand love	138.98		
Brand personality	130.33		
Brand community	104.81		
Customer loyalty	68.38	Purchase intentions	172.56
2010’s			
Co-creation	104.67		
Brand engagement	104.59		
Brand authenticity	46.03		
Online review	47.76		
Content analysis	124.70		
Scale development	173.01		
Structural equation modelling	156.42		
Big data	162.05		
Purchase intention	203.89		
2020–2021			
Covid-19	98.04		
Brand coolness	92.37		
Artificial intelligence	46.49		
Machine learning	55.06		
Sharing economy	196.17		
Virtual reality	168.37		
Sentiment analysis	17.09		
Text mining	93.80		
Technology acceptance model	165.1		
Brand loyalty	213.73		

Only clusters that represent concepts and with scores higher than 20 were considered. *Score*: Shows the relevance value assigned to the cluster

MeaningCloud software uses Text Clustering API that allows to reveal the implicit structure and the meaningful subjects embedded in the contents of the articles. This API takes a set of texts and distributes them in groups (clusters) according to the similarity between the contents of each article. The clustering process (i) uses lemmatization technology (a morphological transformation that changes a word as it appears in text into the base or dictionary form of the word, called lemma, by removing the inflectional ending of the word) to consider all the morphological variants of a term (e.g., high/higher/highest) (Kanis et al., 2010), (ii) allows to define words that should not be considered in the analysis process due to their little semantic relevance, (iii) groups the articles according to their relevance with respect to the context in the analyze and not purely textual similarity, (iv) assigns to each cluster a name which semantically represents its contents (Fan et al., 2006; MeaningCloud, 2022).

This text mining technique was very useful to analyze a large amount of information, it also helped in the elaboration of the framework (presented in Sect. 4) and the suggestions for future research. From the Scopus and WoS databases, it was possible to verify that the first articles on branding stored in them date from the seventies of twentieth century. Through the keywords of the articles, it was possible to identify the core concepts (those most analyzed in the articles) for each decade, allowing a timeline to be drawn on the evolution of the content of the publications.

## Brand journey

**Fig. 3:**
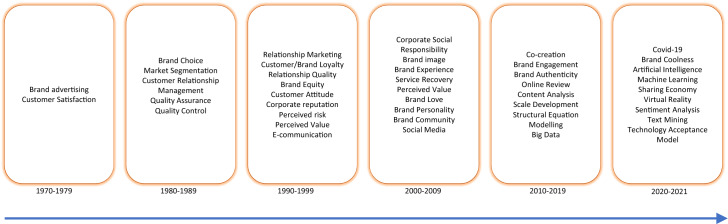
Timeline on the evolution of the content of publications

The timeline gives us an overview of the trends in research of brands (see Fig. 3). Initially the marketing communication of brands emerges as relevant. Researchers and practitioners soon understood the importance of the impact of advertising brands on sales (Gronhaug, 1973; Samuels, 1970) and the determinants of customer satisfaction (Hawes & Arndt, 2007).

The 1980’s witnessed a rapid development of information associated with the customers profile through the direct marketing activities and database information, leading to an effort to delimit the concepts of customer satisfaction, brand loyalty or customer retention, so relevant in the “customer relationship management” (CRM) process (Nevin, 1995). Oliver (1981, p. 27) summarizes the nature of satisfaction as “the emotional reaction following a disconfirmation experience which acts on the base attitude level and is consumption-specific”.

Service quality assurance and control are keywords embedded in the construct of service quality, from where the Servqual’s scale and the Gap model were created to measure service quality (Parasuraman et al., 1985, 1988) or the Grönroos-Gummesson Quality Model, which regards four dimensions of service quality (conception, production, delivery, and relational) (Grönroos, 1990a; Gummesson, 1987). Both satisfaction and service quality perceived by customers and other stakeholders can predict brand loyalty (Reichheld & Sasser, 1990).

The decade is also marked by research intending to understand the consumer behavior, their brand preference and choice (Pitts & Woodside, 1983) and the processes to segment the market to differentiate the preferences, which is also analyzed during the nineties (Dibb, 1992).

In the 1990’s the relationship between firms and their customers focusing on the one-to-one relationships with customers that integrate database knowledge (Peppers & Rogers, 1993), gradually extend to many relationships (Gummesson, 1987). Although the expression “relationship marketing” was born through the voice of Berry (1983), the research on branding in the era of marketing with customers and other stakeholders grew in the last decade of the twentieth century. Relationship marketing is developed when companies/brands “establish, maintain and enhance relationships with customers and other partners, at a profit, so that the objectives of the parties involved are met” (Grönroos (1990b, p. 138). The role of brands in this relational era become more salient representing the identity of the company or products to which the name or term is employed. Expressions such as “attract”, “on-going relationship”, “exchange”, and “interaction”, “relationship quality” are often used (Bagozzi, 1995; Berry, 1995). Relationship quality is “as a higher order concept, implying that a better quality relationship results in a lower level of conflict as well as greater trust, commitment, expectation of continuity, and willingness to invest (Kumar et al., 1995, p. 55).

The increasing role of brands in the consumers’ life drives researchers to become more interested in understanding how consumers relate to brands: consumer-brand relationships. The Fournier’s (1998) brand relationship quality model consolidates and boots the research on the topic. From late nineties, interdependency theory and theories of attraction have been often employed to support the studies. These theories are commonly used in psychology, parent–child relationships (Bowlby, 1979) or adulthood romantic relationships (Hazan & Shaver, 1994). As between two human beings, emotional attachment can occur between human beings and animals, destinations, special objects, brands (Ahuvia, 2005; Price et al., 2000; Schouten & McAlexander, 1995), human brands or celebrities (Thomson, 2006).

Brand equity is another construct that had achieved relevance in the nineties and during the first years of the twenty-first century (Buil et al., 2013; Rangaswamy et al., 1993; Roy & Cornwell, 2003). Diverse studies follow the Aaker’s (1991) perspective which operationalizes the construct as a set of assets (or liabilities) comprising brand awareness, brand associations, perceived quality, brand loyalty and other proprietary assets (Sloot et al., 2005). Keller (1993) considers brand equity as customer-based brand equity, meaning the differential effect of brand knowledge on consumer response to the marketing of the brand.

Not only does brand equity contribute to confer value to a brand, but its reputation and credibility are external signals that can enhance the relationship with consumers (Herbig & Milewicz, 1993) helping them in the moment when choosing what to purchase. A bad reputation may destroy a brand, making its rehabilitation difficult (Herbig et al., 1994). Therefore, brand reputation can reduce the perception of risk associated to the purchase process (Mitchell & Greatorex, 1993). Risks during the purchase process can be perceived by the final consumer, as well as by the organizational client (Mitchell, 1994) and become even more relevant in the e-commerce. The services offered by the brands through the internet become gradually more studied during the twenty-first century (Verhoef & Langerak, 2001; Vroomen et al., 2005).

Perceived value is a valuable concept in branding because it evaluates how consumers perceive a brand through the product (good/service) they receive (Sheth et al., 1991). Perceive value gains even more interest during twenty-first century (Boksberger & Melsen, 2011), with academics differentiating individual, social, utilitarian, hedonic or luxury values (Loureiro & de Araújo, 2014; Ryu et al., 2010).

The first decade of the twenty-first century bears witness to a development of research around the concept of brand image. The Keller’s brand knowledge model (Keller, 2003) marks a tipping point that drive researchers to be interested in how consumers memorize brands, how brand image is encoded in the consumers’ mind. Brand image is, thus, created through the associations connected to attributes, benefits, and positive attitudes that consumers have towards the brand (Keller, 2003). Other similar expressions are, for instance, corporate image, country image (Laroche et al., 2005), or destination image (Gallarza et al., 2002). Corporate social responsibility can contribute to the identity of the brand and consequently enhance the image of the brand, but it represents the firm’s commitment to its societal obligations, which can consider environmental concerns, employees’ satisfaction, no slave work, and attention to society as a whole (Bhattacharya & Sen, 2003, 2004).

Consumer experience reveals to be of economic interest due to the competitive advantage (Pine & Gilmore, 1998) that brands have when offering a differentiated (Lemon & Verhoef, 2016) or a memorable experience (Morgan & Xu, 2009). Pine and Gilmore (1998, p. 98) claim that an experience happens “when a company intentionally uses services as the stage, and goods as props, to engage individual customers in a way that creates a memorable event”. Brand experience expresses the cognitive (analytical and thinking), sensory and affective states of individuals and how they behave towards the brand (Schmitt, 1999). Experiences affect the customer’s emotions and memories and consequently influence brand loyalty (Ha & John, 2010).

In the twenty-first century researchers become more interested in studying the emotional states of consumers (Mattila & Enz, 2002) and service recovery, when something fails and how it can affect consumers cognitively and emotionally (Han et al., 2010; Loureiro et al., 2012; Mattila, 2001; Mattila & Patterson, 2004).

The emotional states of consumers have driven academics to understand the strong ties that consumers can develop toward brands, the so-called brand love (e.g., Carroll & Ahuvia, 2006; Albert et al., 2008; Batra et al., 2008). Albert et al*.* (2008) consider two main components of brand love—passion and affection—based on the interpersonal triangular theory of love (Sternberg, 1986). The two components aggregate six first order dimensions: idealization, intimacy, pleasure, dream, memories, and uniqueness. Batra et al*.* (2008) start to develop the brand love prototype, the precursor of the scale of brand love (Batra et al., 2012) in which self-brand integration, passion-driven behaviors, positive emotional connection, long-term relationship, positive overall attitude valence, attitude certainty, and anticipated separation distress, influence brand loyalty. The second decade of twenty-first century watched a proliferation of publications intending to explore brand love in different contexts (e.g., Gumparthi & Patra, 2020; Kaufmann et al., 2016). Brand hate captured less attention so far (Romani et al., 2015; Zarantonello et al., 2016).

Brand personality construct that has its roots in the idea of anthropomorphism, by attributing human traits to brands (Aaker, 1997). Aaker (1997, p. 347) defines brand personality as “the set of human characteristics associated with a brand”. From this seminal work, other studies attempt to further develop the concept and analyze its antecedents and outcomes (Jani & Han, 2013).

Muniz and O’Guinn (2001, p. 412) present the concept of brand community as a “specialized, non-geographically bound community that is based on a structured set of social relations among admirers of a brand”. Brand community starts to be considered as a customer-customer-brand triad (Muniz & O’Guinn, 2001) to become a “social aggregation of brand users and their relationships to the brand itself as a repository of meaning” (McAlexander et al., 2002, p. 39). This social perspective of brand community opens the doors to incorporate the social media. Brand communities can have both physical and online existence. Concomitantly, other close concepts appear, these more dedicated to niche communities: brand cult (Brown et al., 2003) and brand tribalism (Veloutsou & Moutinho, 2009). The communities can group people who love and support a certain brand or the opposite, constituting anti-brand communities (Romani et al., 2015; Sorensen & Drennan, 2017).

Social media facilitates the process of sharing information and amplifying brand communication (Laroche et al., 2012; Leung et al., 2013). As in physical stores, the stimuli of the webpages are atmospheric cues that influence the online purchase process of brands (Wang et al., 2015). E-influencers and celebrities are themselves brands that use their social network to convey their opinion about the goods and services that they experience (Thomson, 2006).

In the second decade of twenty-first century, academics gradually become more motivated to understand customers through online reviews and using methodological techniques such as content analysis and text mining (Schuckert et al., 2015), particularly sentiment analysis and the use of artificial intelligence (AI) algorithms to treat large amount of data (big data) (Hao et al., 2015).

Brand engagement received more attention from both academics and practitioners since 2010, where behavioral dimensions (van Doorn et al., 2010; Kumar et al., 2010; Verhoef et al., 2010), psychological processes (Bowden, 2009) or even a multi-dimensional approach (Brodie et al*.* 2011; Brodie & Hollebeek, 2011) are considered. The multidimensional approach has been employed in latest studies (Hollebeek et al., 2014; Hwang et al., 2015; Prentice & Loureiro, 2018; Prentice et al*.*, 2019). Engagement occurs by co-creative and interactive experiences of customers or other stakeholders () with a focal agent/object (the brand or the firm) in product/service relationships (Brodie et al., 2011). Thus, the engagement process can support the co-creation of experiences, since co-creation is about the joint creation of value embedded in the personalized experience (Prahalad & Ramaswamy, 2004; van Tonder & Petzer, 2018). Value co-creation can also be mirrored in niche brands created for the purpose of sharing economy, that is, a socio-economic system created to share resources (e.g., peer-to-peer lending, crowdfunding, carsharing, coworking, talent-sharing, house renting) (Fatma et al., 2020).

As with brand engagement, brand authenticity has been considered as multidimensional (Bruhn et al., 2012; Napoli et al., 2014; Schallehn et al., 2014; Moulard et al., 2015). The meaning of brand authenticity is associated with sincerity, innocence, originality and genuineness (Grayson & Martinec, 2004; Guèvremont, 2018; Moulard et al., 2016). Brand authenticity depends on the atmospheric cues of the experience (Beverland & Farrelly, 2010) influencing brand trust and brand loyalty (Lude & Prügl, 2018). Authentic meaning that the brand behaves consistently to its essence or roots (be genuine) (Nancarrow et al., 2003; Sriramachandramurthy & Hodis, 2010) is one dimension of brand coolness (Warren et al., 2019).

Coolness has diverse synonyms (e.g., hip, awesome, chill) and it is “a subjective and dynamic, socially constructed positive trait attributed to cultural objects inferred to be appropriately autonomous” (Warren & Campbell 2014, p. 544). Other dimensions of this multidimensional and complex construct are energetic, aesthetically appealing, original, popular, rebellious, high status, subcultural, iconic, and extraordinary (Warren et al., 2019). Mass cool brands (those recognized by the broader population) are perceived to be mainly energetic (enthusiasm, energy, and vigorous) (Aaker, 1997), high status (prestige, sophistication) (Nancarrow et al., 2003), popular (fashionable and trendy) (Dar-Nimrod et al., 2012) and iconic (cultural symbol) (Warren & Campbell, 2014). Niche cool brands (recognized within small subculture) are more associated with other dimensions, particularly rebellious (fighting or contesting conventions and norms) (Bruun et al., 2016; Nancarrow et al., 2003), original (different, creative) (Bruun et al., 2016), and authentic.

The year of 2020 was quite noticeable by the pandemic situation of Covid-19 and thus brands indirectly suffered with that situation with physical stores, places and destinations closed and consumers afraid to take the risk of revisit them (Jimenez-Barreto et al., 2021). During that timeframe, brands were adapting to the new conditions and the imposition of social distance to avoid contagion. In this condition, the interest for new technologies (virtual reality, augmented reality, artificial intelligence) grows. Brands are adopting AI algorithms to incorporate in diverse platforms to communicate with customers (e.g., chatbots), consumers are using virtual assistances to assist them in their lives, or robots are being used as frontline employees or to treat the large amount of data collected from consumers (Ashfaq et al., 2020; Choi et al., 2019; Kim & Han, 2020; Loureiro et al., 2021).

Analyzing the use of the terms as “structural equation model” in the keywords of the articles, we can claim that the structural equation modelling statistical technique was quite predominant in the first decades of twenty-first century (e.g., Ha & John, 2010; Loureiro et al., 2012). Gradually, researchers are adopting text mining techniques to find patterns in large amount of data (e.g., Loureiro et al., 2019). The studies using new technologies (e.g., VR-virtual reality, AR-augmented reality, AI-artificial intelligence) are still very focused on Technology Acceptance Models (e.g., UTAUT—Unified Theory of Acceptance and Use of Technology, TAM—Technology Acceptance Model).

## Framework for the psychological characteristics of the branding process

**Fig. 4:**
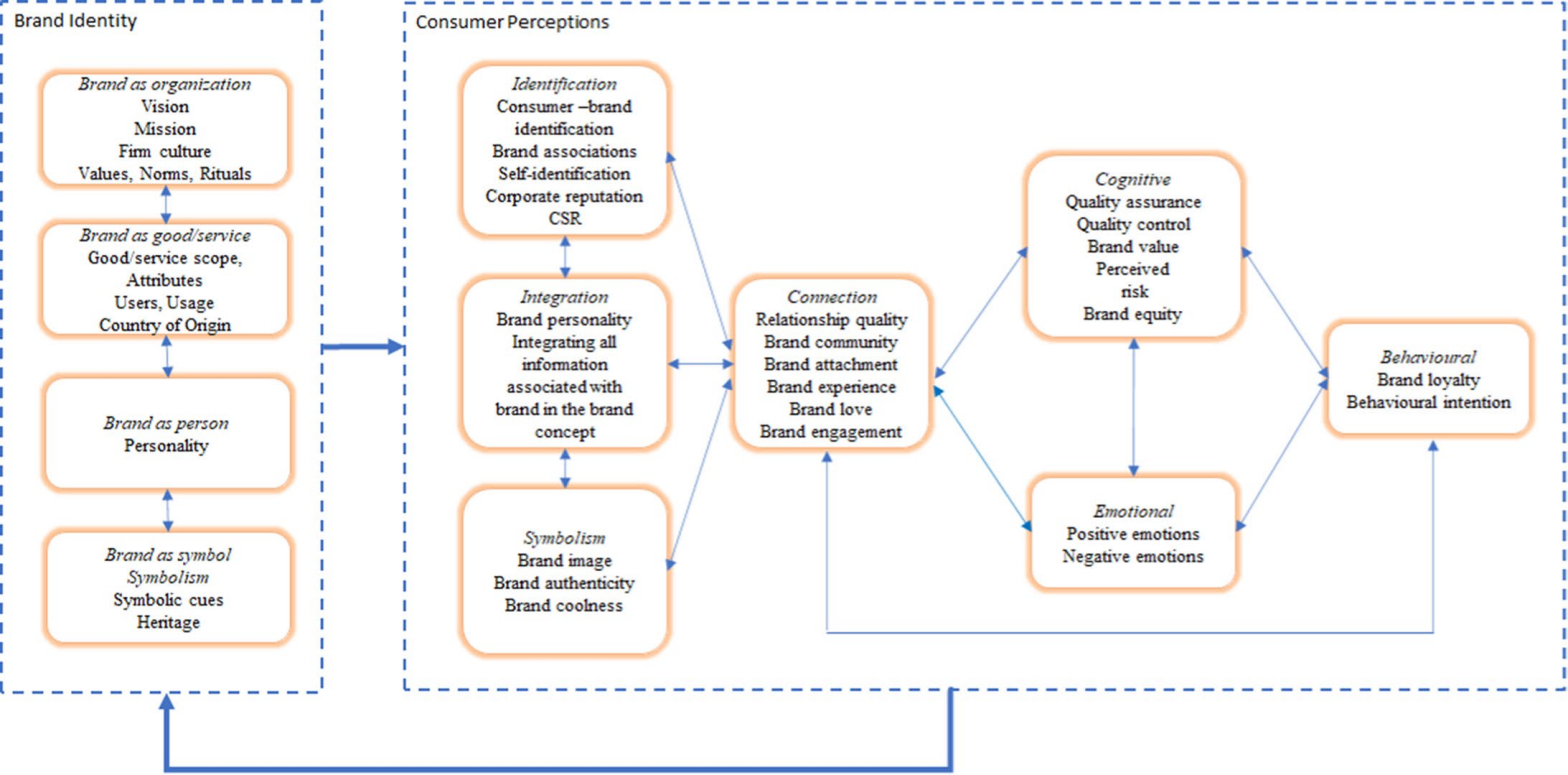
Framework for the psychological characteristics of the branding process

The several decades of publications about brands provided a diversity of concept and constructs. The framework shown in Fig. 4 is composed by two major parts, brand identity and consumer perceptions. The two parts are connected through arrows representing a cycle. The cycle means that information about the brand is communicated to consumers, and they reciprocate by giving their feedback to brands, allowing for continuous improvement. The double arrows represent the associations between the brand characteristics.

Aaker (1991) and de Chernatony and Dall’Olmo (1998) are the first to propose the concept of brand identity. Brand identity has four characteristics, brand as products (good/service) firm, brand as product, brand as person and brand as symbol. Brand as product firm represents the culture of the organization owner of the brand, its values, norms, and rituals. The vision and the mission of the firm should be aligned with the communication policy of the brand. The mission gives the objectives of firm, while the vision is a description of what the firm intend to achieve. Thus, those objectives need to be mirrored in the identity of the brand. Brand as product comprises the realm of features and functions that characterize a product (scope), its characteristics, users, usage, and country of origin (Aaker, 1991; de Chernatony & Dall’Olmo, 1998). For instance, the brand of an hotel chain implemented in diverse countries benefit in being created in a country with a good image among guests. Brand as a person is the human component of a brand, representing the traits of personality of the brand (Aaker, 1997). Consumers are related with brands as if they are other human beings: anthropomorphism (Fournier, 1998). Finally, brand as symbol (e.g., colors, graphism of the name, the figure of animals or other living beings, the heritage of the brand), provide relevant aspects of the brand which contribute to the structure and cohesion of the identity (Liu et al., 2021).

From the consumer’s perception side, seven characteristics are highlighted: identification, integration, symbolism, connection, cognitive, emotional, and behavioral. Identification, integration, and symbolism come directly through the marketing communication of the brand, its signals. Identification represents the process by which consumers identify themselves with the brand/firm asset. Prior studies attempt to conceptualize and analyze constructs such as: brand associations, self-identification, or corporate social responsibility (Keller, 1993, 2003). Corporate reputation and corporate social responsibility (CSR) are two concepts that mirror relevant information about brands and their positioning in the market (Bhattacharya & Sen, 2003, 2004; Mitchell & Greatorex, 1993). All kind of associations created in the consumers’ mind—from those more related to the characteristics of the product or those associated with the users of the brands—are relevant to their knowledge.

Integration means the information perceived and interpreted by consumers based on the data spread by the brand through the marketing communication campaigns. Brand personality (Aaker, 1997; Jani & Han, 2013) is a construct embedded in the integration characteristic. Consumers can identify with a certain brand because they perceive similar traits of personality between them and the brand (Ong et al., 2017). Consumers aggregate all the information received and perceived about a certain brand to build their own knowledge about it.

Symbolism means that brands can represent groups and cultures. Diverse brand are cultural icons (e.g., Harley-Davidson, Coca-cola) and represents nations, or generations. Thus, symbols contribute to enhance the image of the brand in consumers’ mind, but they are also associated with constructs such as brand authenticity (contribute to the perception of genuine and original) (Beverland & Farrelly, 2010; Bruhn et al., 2012) and brand coolness (through dimensions as original, iconic, authentic, or rebellious) (Warren et al., 2019). Hence, brand coolness is deeply associated with symbols that consumers perceive in a brand.

Connection is the process by which brands, and consumers exchange ideas and information. Several constructs deal with the concept of connection, such as relationship quality (with the core concepts of trust, commitment, and satisfaction), brand community (McAlexander et al., 2002; Muniz & O’Guinn, 2001) (the group where consumers interact for the purpose of the brand), brand attachment (emotional connection) (Thomson, 2006), brand love (Batra et al., 2012) (represents a long-term relationship with a brand, an emotional attachment, the willingness to invest resources and the anticipated separation distress). Concepts as brand experience (Brakus et al., 2009) and brand engagement (Brodie & Hollebeek, 2011) have several dimensions, combining emotional, cognitive, and behavioral elements, but in their core, they translate the relationship between brands and consumers (Bilro et al., 2018).

The cognitive characteristic includes more traditional constructs to measure the quality and value of a brand, such as: quality assurance, quality control, brand value, perceived risk, or brand equity (Buil et al., 2013). Emotional represents all sort of emotions regarded in previous studies, such as pleasure, arousal, delight, disgust, boredom (Oliver et al., 1997). The behavioral characteristic considers the constructs usually regarded as outcomes, such as brand loyalty and behavioral intentions (Laroche et al., 2012; Loureiro et al., 2012; Roy & Cornwell, 2003).

In the side of the consumer perceptions, identification, integration, and symbolism are in the same level and are drivers to the more cognitive and emotional characteristics of the brands, through connection. When consumers identify themselves with a certain brand, integrate the brand in their lifestyle and perceive it as a symbol, they will tend to be more connected. The connection process leads consumers to interiorize the stimuli received from the brand and to develop cognitive and emotional states of appraisal. Finally, consumers will behave depending on those cognitive and emotional states developed.

## Future trends

The overview of gaps and future trends on brand is presented based on theory, themes and context, psychological characteristics, and methodology (Terjesen et al., 2016) (see Table 2).

**Table 2:** Research suggestions

Research domain		Major gaps	Research questions
Theory		Lack of unified theories for brand identity that also consider no-brands Lack of theories that integrate the physical, online, and virtual services Lack of theories that can regard humanized robots as customers	What will be an integrated theory of brand identity? Could such theory integrate the physical, online, and virtual service? Can the brand knowledge model be extended to the virtual service? What could change when customers are non-humans (e.g. AI algorithms incorporated or not in humanized robots)?
Themes and context		Lack of studies on no-brands More studies on consumer-brand relationship, particularly the negative site of relationships Lack of studies dedicated to analyzing positive and negative relationships with brands and other stakeholders besides consumers Lack of longitudinal studies tracking the evolution of brand due to the Covid-19 situation Lack of studies on the consumers’ decision process in an era of continuous increment in technology More studies on how brand identity and brand knowledge in different cultural contexts	What strategies and tools are recommended to no-brand? How the continuous increment in technology can affect the consumers’ decision process? how to measure brand value and how monetarize brand health in an era of continuous increment in technology? How technologies enable the co-creation of the brand? How technologies help integrate consumers value and brand? How different can be the marketing communication of well-known and not well-known brands? What are the consequences for brands of Covid-19 situation over time? How manage Ai system that operate as brands (e.g. Alexa, Siri)? And what about when AI systems (embodied in anthropomorphic robots) will operate as customers (for instance in interactions with other stakeholders) What is the perspective of brand identity in less studied cultural contexts? What about brand knowledge?
Characteristics	Cognitive	Lack of understanding the perceptions of service quality, value and risk of brands and no-brands operating with different levels of technologies and AI systems	What relevance will be conferred to service quality with the development of the virtual and artificial contexts? What about the perception of value and risk?
	Identification	Lack of understanding the identification with brands in virtual worlds More research on strategies for brand extensions and co-branding	What associations with brand will be more affective to create identification in the virtual worlds? Can the associations towards the brand be perceived differently in virtual services than in physical and online ones? What about the strategies of brand extensions and co-branding? How does the identification process occur with consumers and no-brands?
	Symbolism	Lak of studies on symbolic cues in the context of virtual world and AI systems Lack of studies on brand coolness	What symbolic cues can brands transfer in the virtual worlds and AI systems? What symbolic cues are expressed by no-brands? How can dimensions of coolness such as iconic, authentic, aesthetical appealing be symbolic cues in online and virtual services?
	Integration	Lack of studies on the personality of brands and no-brands in virtual and AI systems context More studies to understand how humans integrate the information associated with brands from different sources	What traits of personality will prevail in virtual brands? Or on no-brands? Will AI Robots have different personalities? How will humans and AI systems be integrated in the future? Can they (AI systems) become influencers, as human celebrities, and e-influencers? How do consumers integrate the information associated with the brands (different types of service brands) (e.g. lifestyle, innovation, emotional states) and store it in their memory?
	Connection	More studies to understand how brands/no-brands can interact with customers or between brands and non-humans More studies about attachment/avoidance, love/hate, engagement with brands/no-brands and humans in different environmental contexts	How do the human-virtual brands connect? What about when the connection is stablished between brands and non-humans (e.g. AI robots)? How can we extend the attachment/avoidance process to the virtual brands? What about no-brands? Can consumers develop love/hate relationships with virtual service brands? What about with AI algorithms and robots? What about no-brands? How will the evolution of AI systems contribute to the engagement process with brands?
	Emotional	Lack of studies on positive/negative emotional states in difference experience context simultaneously Lack of studies on how AI systems will evolve emotionally and how this is reflected in interactions	How will both positive and negative emotional states be developed between brands and consumers in worlds where consumers experience simultaneously physical, online, and virtual services? With the acceleration of innovation due to the virtual, artificial and nano-technologies, how will consumers and brands evolve emotionally?
	Behavioural	Lack of studies analysing co-creation processes in different types of services, extended to virtual environment and interactions with AI systems Lack of studies on behavioural intentions and actual behaviour comparing virtual and online environments	How will humans behave with no-brands? Will the interactive engaged process and co-creation depend on the type of service brand? If yes, what are the differences? What about other aspects of behavioural engagement? Can humans be more willing to purchase more, pay more or recommend more in the case of virtual brands than online ones? Under what circumstances can it happen?
Methodology		Lack of more exploratory studies (qualitative and mixed approaches) that allow the development of new concepts and frameworks Extensive use of symmetrical analysis of data and lack of asymmetrical analysis Develop new data collection techniques and algorithms to treat them (for instance using AI algorithms) More research on how VR, AR and AI evolve and how to develop research to contribute to brand management and advertising	How will the text mining techniques capable of analysing both qualitative and quantitative data evolve? What other forms of collecting data using surveys will allow a better generalization of the findings? What other techniques of organizing, clustering, and categorizing data will emerge? How will the virtual and augmented technologies evolve? How will the evolution of neuro-techniques conduct us to have more knowledge about the interaction human-non-humans-brands?

### Theoretical domain

Although prior researchers—such as David Aaker, de Chernatony and Keller—have devoted their time to creating models of identity, equity and knowledge of a brand, the proliferation of diverse type of brands and the extension of products to online or virtual, contexts (related to immersive experiences lives in virtual worlds or mixed realities, for instance virtual, augmented reality and mixed reality) call for an integrated theory of identity and knowledge of a brand. Regarding the designated no-brands, they tend to be appealing for certain consumer segments. More than bringing theories and frameworks from other fields of knowledge (e.g., psychology, sociology, economy), future studies should develop specific theories for brands in an environment of intense technological transformation—particularly with the gradual implementation of AI systems and robots—and the emerging trend to create no-brands.

### Themes and contexts domain

New themes are emerging that deserve attention, one is no-brands. No-brands also use names or symbols to represent products, but their managers do not intend to use the traditional tools for positioning, targeting and communicate them. Therefore, what new tools and techniques are being used or should be recommended?

Consumer-brand relationship is a theme that deserves further attention. Although relevant concepts and constructs are associated with the interaction between brands and consumers—for instance, brand love, brand commitment, interdependence, brand hate, brand engagement –researchers should now be more focused on the negative side of relationships, what is and how to handle dysfunctional brand relationship, how to re-establish relationships. In this perspective, other concepts and constructs can emerge, as brand mythology, brand civility, and other relational aspects, as despair (hope), pessimism, pathology, power, victimhood and vulnerability, or cruelty and inhumanity, or equivocation, misunderstanding of relationship rules and templates, and conversational dilemmas. From a more positive site of brand relationships, brand coolness is still a recent and very complex concept that needs future attention to properly understand how to operate the different dimensions in different cultural context and how it evolves with time and lifestyles.

Another gap is found in the lack of studies dedicated to analyzing positive and negative relationships with brands and other stakeholders besides consumers. Although diverse studies focused on brand with other stakeholders not directly involving consumers (e.g., Boyle, 2007; Charters & Spielmann, 2014; Zhang et al., 2015), consumers have deserved the attention of most studies. Regarding the brand marketing communication with different stakeholders, it will be relevant to understand how brand campaigns and messages can be integrated across platforms and devices. Well-known brands and no-brands are expected to have different ways to communicate, but brand managers benefit from more research that clarifies the differences.

The gradual incorporation of technology (e.g., VR, AR, AI)—particularly the AI systems and robots with AI—boosted by the pandemic situation of Covid-19, has led to a shift in the way brands are managed by the owners within the organizations and the way they are perceived by the consumer. Thereby, new research avenues are emerging, regarding Covid-19 and the contribute of technologies to the co-creation of brands and their experiences. As for the specific theme of Covid-19 and its consequences on brand, longitudinal studies are needed in the future. Considering the technologies, consumers and other stakeholders are interacting with brands through AI systems that operates as frontline employees or stewardship of the brand (e.g., chatbots or robots). AI analytical systems can process large amount of data allowing the extreme segmentation of the market and going to micro-targeting and personalization. But how can this continuous increase in technology affect the consumers’ decision process? What about the interaction between AI systems that are itself brands (e.g., Alexa, Siri) with consumers and their families? In this transformation operated by technologies it will also be relevant to investigate how to measure brand value and how monetarize brand health.

In terms of context, the frameworks, constructs, and concepts on branding were developed and analyzed in the Western and Asian contexts. The studies that come from researchers located in the African continent and Latin America are fewer in number. Research that intends to compare different cultural contexts is also recommended. Indeed, consumers in different cultural context will have different beliefs, values and lifestyles leading to different perceptions and desires toward the brands. Regarding the designated no-brands, they tend to be appealing for certain consumer segments. Researchers have an opportunity to study the psychological effect of these no-brands in consumer behavior. What is different in the way they communicate, their signals and how they are perceived by consumers. How the existent frameworks and theories works or not for no-brands contexts. As certain possessions, things, animals, or brands can be seen as part of consumers selves, even in the digital world (Belk, 2013), it is expected that the consumer develops emotional relationships with AI systems and AI robots, particularly when they develop more intuitive and empathic skills (Huang & Rust, 2018).

### Characteristics domain

As for the psychological characteristics of the brands perceived by consumers, these aggregate the consumers’ perceptions and processes related to brands. Cognitive perceptions of brands had its major attention in the last decade of twentieth century and beginning of twenty-first century. Yet, more studies can be performed to understand how consumers perceive the quality and satisfaction or risks of no-brands, or the good/service in virtual worlds and AI systems.

Consumers identification with a brand is key in the decision process to select a product. Therefore, the specific associations that consumers develop in virtual worlds or those developed with no-brands are still an avenue that need to be explored.

Brands display symbolic cues that capture the interest of consumers when they are in line with their value systems and lifestyle. This happens, for example, when brands are perceived to be “cool” (Warren et al*.*, 2019). These symbolic cues should be better analyzed in different cultural and virtual contexts. Brand coolness is a complex construct deserving further attention to properly delimitate the dimensions that are perceived as more relevant depending on the culture and personality of the consumers.

Brand personality has deserved attention from researchers and brand managers for physical and online services, but not studied in the context of no-brands and virtual worlds. These traits of personality communicated by the brands together with other information cues are integrated in the consumers’ mind, creating memories. This phenomenon is still not well-understood, despite efforts made with neuro-techniques and eye-tracking. Thus, the combination of these and other techniques is used to understand the phenomenon of how the consumer interprets and integrates the information.

Connection is largely associated with attachment and engagement (Chang et al., 2021). Although we can find studies dedicated to situations when brands and consumers are together, cooperating, few studies try to understand negative engagement, that is, when consumers are actively downgrading a certain brand. More studies are needed to understand negative relationships with brands, brand hate and the phenomenon of brand avoidance.

Positive and negative emotional states will be special relevant as AI systems and robots develop emphatic skills and eventually become themselves not only brands (or working for brands), but consumers. Aligned with this observation, we may witness an evolution of the purchase decision process and consumer behavior with the proliferation of AI systems in the workplace.

### Methodological domain

In methodological terms, past decades tended to employ a quantitative approach (with massive use of structural equations modelling) (e.g.Chang et al., 2021; Fritz et al., 2017; Jani & Han, 2013; Prentice & Loureiro, 2018), with the increase of information to process, researchers have turned to the use text mining techniques. In the future, it is expected that more tools of text mining will appear and will be used more often. Experiments using neuro-techniques, eye-tracking, virtual, augmented and extend reality for instance will be used more often. Qualitative and mixed approaches are recommended to develop new theories and constructs. Qualitative studies allow to explore new themes and situations, which can be consolidated through the collection and analysis of quantitative data. The symmetric analysis of quantitative data through multiple regression and structural equations modelling is still recommended but should be complemented with the asymmetrical analysis (e.g., fsQCA).

## Concluding thoughts

This overview of the extant literature identifies important steps in the progress of the research on brand. Thus, earlier studies started to be more dedicated to brand choice, quality assurance, brand equity, perceived risk, or corporate reputation. In the late 1990’s the concerns about the relationship between brand and consumers become more relevant. In the twenty-first century, the studies on the psychological consumer perception of brands become gradually more frequent, such as: brand personality, brand experience and brand love. In those days, social media and e-commerce shifted the focus on physical goods/services to online ones. Brand engagement and co-creation both online and offline are key words for academics and brand managers. More recently, brand authenticity and brand coolness are relevant to better understand the preference of consumers. As brands are conquering virtual and artificial contexts, the studies on this topic gain popularity.

The academic audience can benefit from this research by having a summary of the literature and suggestions for future research. Hence, this article offers a timeline on the evolution of the content of publications, where it is possible to observe the most analyzed concepts in each decade. Thus, this article provides readers with an overview of how concepts, meanings and trends in branding have evolved over time. Secondly, academics can use the framework for the psychological characteristics of the branding process in their research, where it is possible to understand the associations between core branding concepts. Thirdly, Table 2 can be a guide for potential further research on the topic.

Practitioners can find here important key well-documented brand models and tools, which can inspire them to create or re-organize brands that can be more emotionally attractive, meaningful, engaged or even regarded as “cool” by consumers. This article will assist marketers in understanding the links between the concepts, helping them to prepare their strategies. For example, marketeers should be aware that to manage a brand there are four interrelated components to be aware to create and develop the identity system of a brand. Practitioners can strategically analyze how they are handling their brands through a self-diagnostic taken into consideration those four components: Brand as organization, brand as product (good/service), brand as person and brand as symbol.

From the customers perspective, marketeers can prepare surveys using scales already developed by academics that can measure the psychological characteristics of the brands, which are positioned on the right side of the framework (Fig. 3). For example, to measure the level of attachment between customers and the brand (brand attachment), marketers can select a study about the subject mentioned in the article to get the scale to be used. Marketers can also understand how the psychological characteristics of the brands are related and which ones directly and indirectly lead to loyalty. Finally, this analysis highlights mainly the evolution of the brand in studies more devoted to the relationship business-consumer rather than business-to-business. The reason lies in a large number of articles that analyze brand issues taking the perspective of consumers in the relationship consumer-brand compared to articles that intended to study brands and their implications from the business-to-business perspective. In the future other studies can be dedicated to business-to-business relationship.
